# Effects of Fractionated Radiation Exposure on Vimentin Expression in Cervical Cancers: Analysis of Association with Cancer Stem Cell Response and Short-Term Prognosis

**DOI:** 10.3390/ijms24043271

**Published:** 2023-02-07

**Authors:** Irina Zamulaeva, Olga Matchuk, Elena Selivanova, Liana Mkrtchian, Anna Yakimova, Victoria Gusarova, Nikita Lipunov, Liudmila Krikunova, Sergey Ivanov, Andrey Kaprin

**Affiliations:** 1 Department of Radiation Biochemistry, A. Tsyb Medical Radiological Research Center—Branch of the National Medical Research Radiological Center of the Ministry of Health of the Russian Federation, Korolev Str.-4, Obninsk 249036, Russia; 2 Department of Radiation and Combined Treatment of Gynecological Diseases, A. Tsyb Medical Radiological Research Center—Branch of the National Medical Research Radiological Center of the Ministry of Health of the Russian Federation, Korolev Str.-4, Obninsk 249036, Russia; 3 A. Tsyb Medical Radiological Research Center—Branch of the National Medical Research Radiological Center of the Ministry of Health of the Russian Federation, Korolev Str.-4, Obninsk 249036, Russia; 4 Department of Urology and Operative Nephrology, RUDN University, Miklukho-Maklaya Str.-6, Moscow 117198, Russia; 5 National Medical Research Radiological Center of the Ministry of Health of the Russian Federation, Korolev Str.-4, Obninsk 249036, Russia

**Keywords:** cancer stem cells, cervical cancer, HeLa, SiHa, epithelial-mesenchymal transition, vimentin, ionizing radiation, radiosensitivity, flow cytometry

## Abstract

Elucidation of the mechanisms for the response of cancer stem cells (CSCs) to radiation exposure is of considerable interest for further improvement of radio- and chemoradiotherapy of cervical cancer (CC). The aim of this work is to evaluate the effects of fractionated radiation exposure on the expression of vimentin, which is one of the end-stage markers of epithelial-mesenchymal transition (EMT), and analyze its association with CSC radiation response and short-term prognosis of CC patients. The level of vimentin expression was determined in HeLa, SiHa cell lines, and scrapings from the cervix of 46 CC patients before treatment and after irradiation at a total dose of 10 Gy using real-time polymerase chain reaction (PCR) assay, flow cytometry, and fluorescence microscopy. The number of CSCs was assessed using flow cytometry. Significant correlations were shown between vimentin expression and postradiation changes in CSC numbers in both cell lines (R = 0.88, *p* = 0.04 for HeLa and R = 0.91, *p* = 0.01 for SiHa) and cervical scrapings (R = 0.45, *p* = 0.008). Associations were found at the level of tendency between postradiation increase in vimentin expression and unfavorable clinical outcome 3–6 months after treatment. The results clarify some of the relationships between EMT, CSCs, and therapeutic resistance that are needed to develop new strategies for cancer treatment.

## 1. Introduction

Radio- and chemoradiotherapy are the main methods for the treatment of cervical cancer (CC), which is the second most frequent gynecological malignancy worldwide and a considerable cause of mortality among females due to adverse results of treatment [1,2]. The development of CC resistance to radiation exposure involves multiple mechanisms, one of which can be related to cancer stem cells (CSCs). Accumulating evidence for various solid tumors demonstrates high resistance of CSCs to low linear energy transfer (low-LET) ionizing radiation and many antitumor drugs in vitro and in vivo; therefore, it is suggested that CSCs can persist after antitumor therapy and lead to disease recurrence and progression in a part of patients [3,4,5]. 

We have shown previously a wide individual variability in quantitative changes in the CSC pool after the first sessions of radiation therapy of CC patients and the predictive value of the CSC radiation response for short-term results of treatment (tumor regression degree 3–6 months after treatment) [6]. Elucidation of mechanisms of the CSC response to fractionated radiation exposure is of considerable interest for further improvement of anticancer therapy. As shown for a single radiation exposure, the epithelial-mesenchymal transition (EMT) of cancer cells plays an important role in postradiation formation of the CSC pool [7,8,9,10,11], but the relationship of these processes under fractionated irradiation has not been sufficiently studied, especially in CC patients. Although the activation of the EMT program is associated with acquisition of stem-like characteristics in different cancers, recent studies have presented a more nuanced understanding of the relationship between EMT and CSCs [12,13,14,15]. The dynamics of EMT, especially its last stages, is of considerable interest, since this process often remains incomplete in tumor cells, and the clinical significance of complete EMT has not been clearly elucidated [16]. One of the key end-stage markers of EMT is vimentin, which appears to be at the center of signaling pathways that control plasticity and migratory capacity of cancer cells, resistance to therapy, and adaptation to changes in the microenvironment [14]. Elucidation of patterns of postradiation expression of this protein, individual features of changes in the expression of vimentin during radiotherapy of patients, and their relationship with the formation of a pool of CSCs could provide clues to developing novel therapeutic strategies or combination therapy regimens for CC treatment, including personalized approaches to anti-EMT therapy. The aim of this work is to evaluate the effects of fractionated radiation exposure on the expression level of vimentin, which is one of the EMT end-stage markers in cervical cancers in vitro and in vivo, including the association of postradiation changes in vimentin expression with CSC radiation response and short-term prognosis. 

Human papillomavirus (HPV) infection is known to be the main etiological factor in squamous cell CC. Given the known differences in the radiosensitivity of HPV 16- and HPV-18 positive CC cells, it was interesting to study the relationship between CSCs and postradiation expression of vimentin among different HPV genotypes. We used established cell lines infected with HPV of the main high-risk genotypes, which are present in squamous cell CC in a total of more than 85% of cases: HPV 18-positive line HeLa and HPV 16-positive line SiHa. The CSC proportions were assessed using flow cytometry in HeLa and SiHa cultures after γ-radiation exposure using a conventional regimen of dose fractionation (2 Gy/day) at total doses (TDs) of 2, 4, 6, 8, and 10 Gy. The mRNA and protein expression of vimentin was determined using real-time polymerase chain reaction (PCR) assay and flow cytometry after irradiation at the same TDs. The scrapings from the cervix of 46 CC patients were examined to determine the proportions of CSCs, expression of vimentin, and stemness-related genes before treatment and after irradiation at a TD of 10 Gy during conventional radio- or chemoradiotherapy. This TD was chosen in our study as the prognostic value of the CSC radiation response was previously shown for this dose [6].

## 2. Results

### 2.1. Changes in the CSC Number after Fractionated Radiation Exposure

Two methods were used to identify CSCs by flow cytometry: immunophenotyping based on the analysis of cell surface markers and evaluating the efflux efficacy of the fluorescence dye Hoechst 33342 (Ho342) from cells. CSCs are known to have the ability to pump out this dye due to the high expression of ATP-binding transporters and form a so-called side population (SP) in contrast to non-CSCs. Identification of CSCs in HeLa and SiHa cell cultures was performed using an SP assay. In addition, the expression of CSC-associated marker CD133 was assessed in SiHa cells, and the proportion of CD133^+^ cells was determined. CSCs in cervical specimens from CC patients were identified by CD44^+^CD24^low^ immunophenotype.

**Figure 1:**
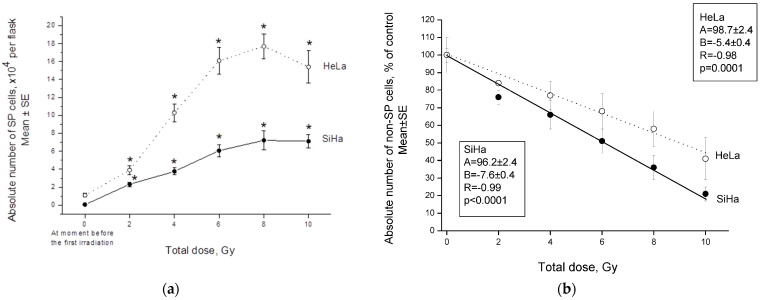
Effects of fractionated γ-radiation exposure on the absolute number of side populations (SP) (**a**) and non-SP cells (**b**) of HeLa and SiHa lines 24 h after the last radiation exposure. The parameters of the linear regressions Y = A + BX are indicated, where X is a total dose (TD) of γ-radiation, Y is the absolute number of non-SP cells of the corresponding cell lines. * *p* < 0.001 in comparison to the initial level before the first irradiation.

The proportion of SP cells in unexposed HeLa and SiHa cultures varied significantly, averaging (±SE) 5.40 ± 0.60 and 0.25 ± 0.02%, respectively. After irradiation, there was an increase not only in the proportion of CSCs, as shown earlier for both cell lines [17], but also a multiple increase in the absolute number of CSCs with an increase in a TD up to 8 Gy (Figure 1a). Statistically significant differences in the CSC absolute number were found in both cell lines after irradiation with the first dose fraction in comparison to that before irradiation. The absolute number of non-CSCs (non-SP cells) decreased with radiation dose in comparison to the unexposed control (Figure 1b), as expected.

**Figure 2:**
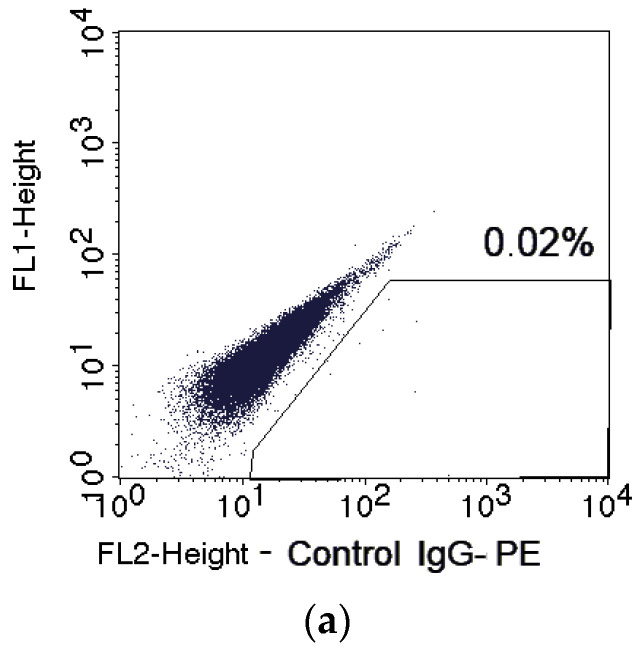

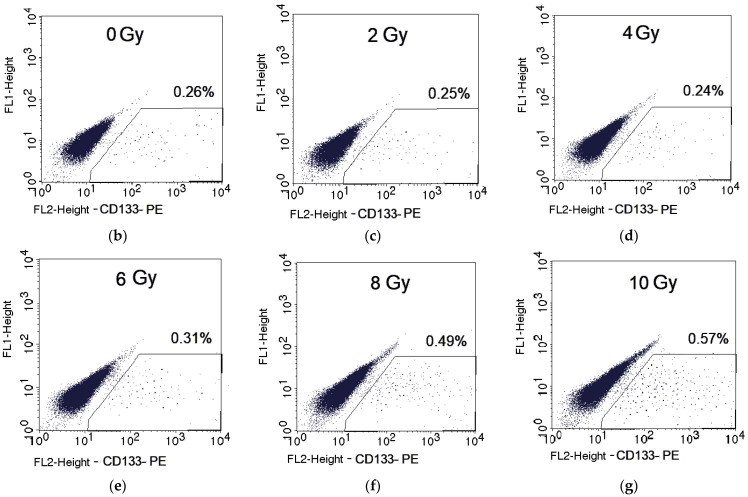
Representative dot plots for fluorescence of SiHa cells in the control of nonspecific binding (**a**) and after staining of unexposed (**b**) and exposed samples to fractionated γ-radiation at various TDs (**c**–**g**) with phycoerythrin (PE)-labeled antibodies to CD133. The region of CD133^+^ cells is highlighted and their percentage is indicated in each plot.

**Figure 3:**
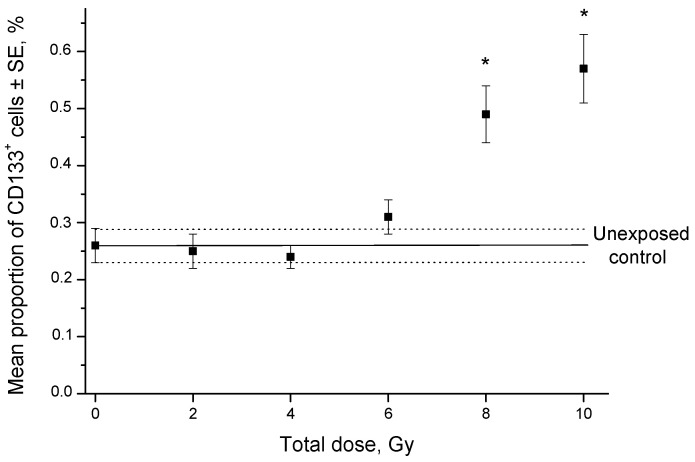
Effects of fractionated γ-radiation exposure on the proportion of CD133^+^ cells in SiHa line cultures 24 h after irradiation. The solid horizontal line indicates the mean proportion of CD133^+^ cells in unexposed samples with standard error (SE, dotted lines). * *p* < 0.05 in comparison to unexposed cells.

The proportion of CD133^+^ cells was higher after irradiation at doses of 8–10 Gy than in the control (Figure 2 and Figure 3).

**Figure 4:**
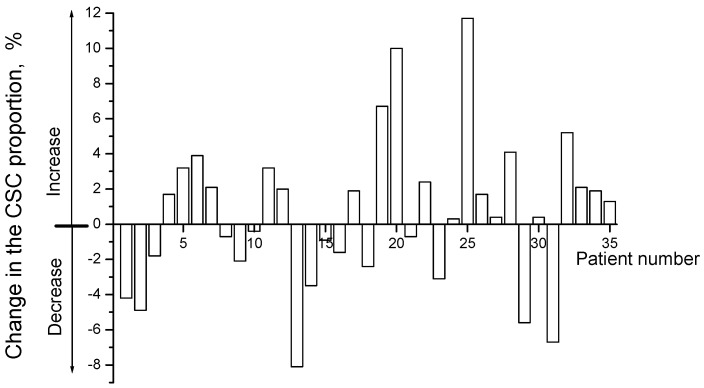
Changes in the proportion of CD44^+^CD24^low^ cancer stem cells (CSCs) in cervical scrapings from 35 cervical cancer (CC) patients 24 h after radiation exposure at a TD of 10 Gy. Positive values indicate an increase in the proportion of CSCs after irradiation, and negative values indicate a decrease in this indicator.

The average proportion of CD44^+^CD24^low^ CSCs in cervical scrapings from CC patients was 3.3 ± 0.5% before treatment and 3.9 ± 0.5% after irradiation at a TD of 10 Gy (*p* = 0.44). The CSC proportion increased in 20 patients (57.1%) after radiation exposure and decreased in the other 15 patients (42.9%) (Figure 4). The average increase in the CSC proportion in the first subgroup was 3.3 ± 0.7%, and the average decrease in this indicator in the second subgroup was 3.1 ± 0.6%.

### 2.2. Radiation-Induced Changes in Vimentin Expression

**Figure 5:**
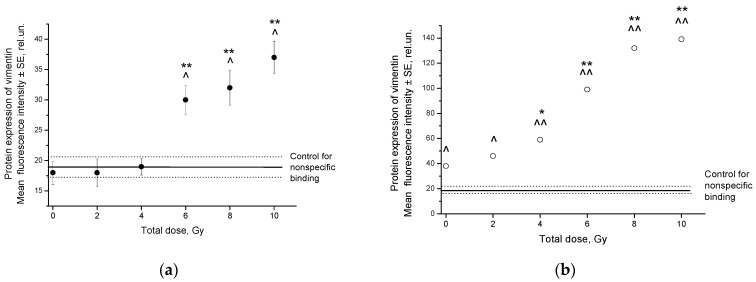
Protein expression of vimentin in SiHa (**a**) and HeLa (**b**) cells as determined using flow cytometry after staining exposed and unexposed cells with labeled antibodies to vimentin and snail hemocyanin (control for nonspecific binding). * *p* = 0.02 in comparison to unexposed cells. ** *p* < 0.001 in comparison to unexposed cells. ^ *p* < 0.01 in comparison to control for nonspecific binding. ^^ *p* < 0.001 in comparison to control for nonspecific binding.

Protein expression of vimentin increased after irradiation of SiHa cells at TDs of 6–10 Gy, and HeLa cells at TDs of 4–10 Gy in comparison to that in unexposed cells, as shown by flow cytometry of cells stained with labeled antibodies to this protein (Figure 5). The maximum increase in fluorescence intensity was 18 relative units (rel.un.) in SiHa cells and 101 rel.un. in HeLa cells after radiation exposure compared to unexposed cells. The level of vimentin protein expression increased 6.3-fold in exposed HeLa cells in comparison to that in unexposed cells: 120 vs. 19 rel.un. after subtracting nonspecific binding, respectively. Meanwhile, the expression of this protein was not found in unexposed SiHa cells, since the fluorescence intensity of cells with labeled antibodies to vimentin corresponded to that in the control for nonspecific binding. In contrast, unexposed HeLa cells expressed vimentin rather intensively. Moreover, the pretreatment expression of vimentin in SP cells was 1.5 times higher than in non-SP cells, as shown by laser scanning microscopy of pre-sorted HeLa cells. Thus, the average fluorescence intensity of SP cells was 30.0 ± 1.0 rel.un., and this indicator for non-SP cells was 19.6 ± 1.2 rel.un. (*p* < 0.001) after preliminary subtraction of fluorescence due to nonspecific binding. 

It is interesting to note that the protein expression of vimentin after irradiation at a dose of 2 Gy did not differ from that in unexposed cells of both lines. At the same time, the absolute number of SP cells increased significantly after radiation exposure at the same dose.

**Figure 6:**
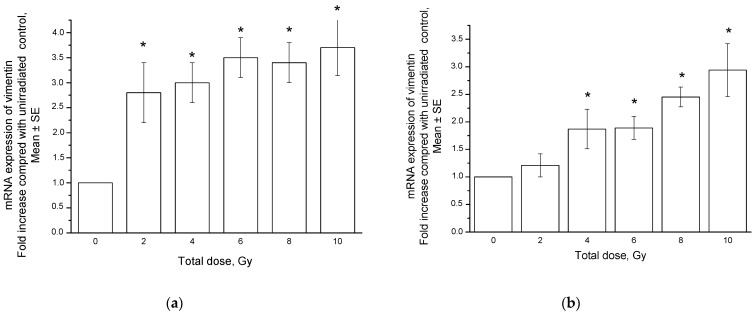
Vimentin mRNA expression in SiHa cells 24 h (**a**) and 8 days (**b**) after fractionated radiation exposure. * *p* < 0.01 in comparison to unexposed cells.

The mRNA expression of vimentin in SiHa cells increased significantly 24 h after each dose fraction in a range of 2–10 Gy compared to that in unexposed cells (Figure 6a). The dose-dependent increase in mRNA expression persisted in the TD range of 4–10 Gy for 8 days after irradiation (Figure 6b), although it slightly decreased compared to the short-term effects at 24 h.

**Figure 7:**
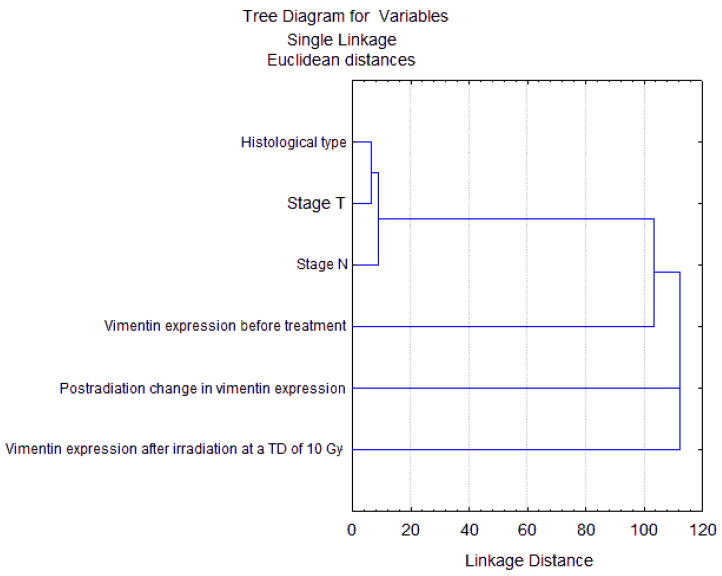
Hierarchical clustering dendrogram of CC patients using 6 variables, including the main clinical and morphological parameters and data on vimentin expression assessed by proportion of vimentin-positive cancer cells before and after radiation exposure at a TD of 10 Gy (*n* = 35). Clinical and morphological parameters are closely associated with each other (Euclidean distances < 10), but not with vimentin expression (Euclidean distances > 100).

The average proportion of vimentin-positive cancer cells was 14.7 ± 2.2% in the group of 35 patients before treatment. This indicator increased significantly to 26.9 ± 2.8% after radiation exposure at a TD of 10 Gy (*p* = 0.001). Table 1 shows data on vimentin expression in subgroups of patients with different clinical and morphological parameters of disease (stage, status of lymph node involvement, and histological type) and treatment (radio- and chemoradiotherapy). No significant association between these parameters or treatment methods and vimentin expression was found either before treatment or after irradiation. The results of the cluster analysis confirmed the conclusion that there was no association of vimentin expression with clinical and morphological parameters (Figure 7).

**Figure 8:**
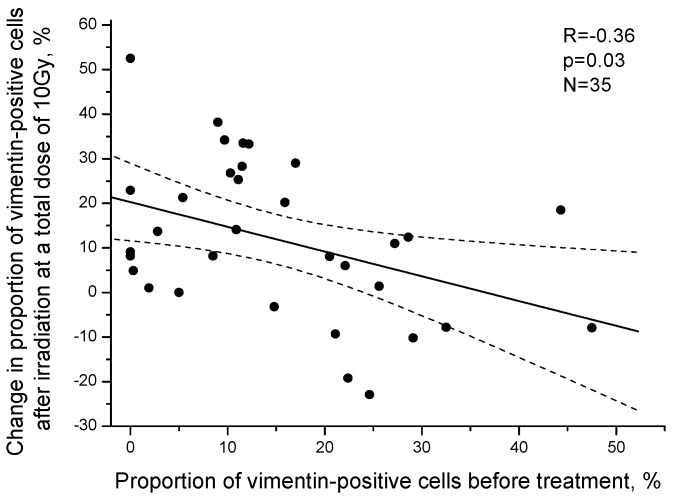
Correlation between the proportion of vimentin-positive cancer cells in the cervical scrapings from CC patients before treatment and the change in this indicator after irradiation at a TD of 10 Gy. Positive values on the *Y* axis indicate an increase in the proportion of vimentin-positive cells after irradiation, whereas negative values indicate a decrease in this indicator. The dotted lines indicate 95% confidence limits for linear regression (solid line).

An inverse correlation of postradiation changes in the proportion of vimentin-positive cancer cells with the initial number of these cells before treatment was revealed (R = −0.36, *p* = 0.03) (Figure 8).

**Figure 9:**
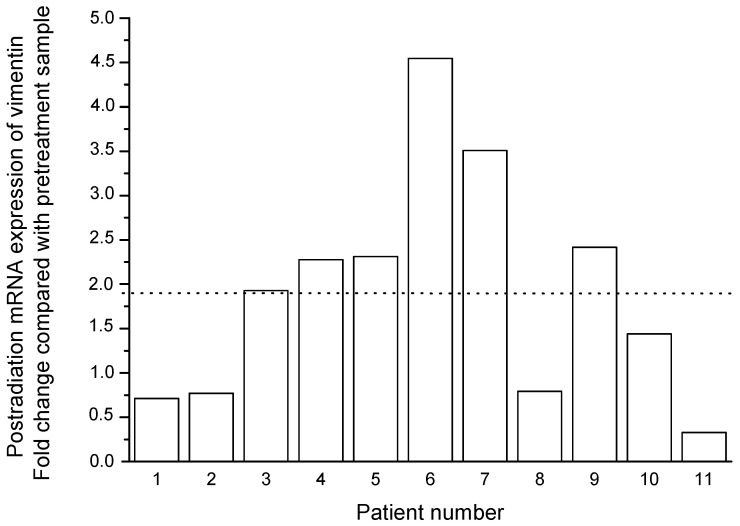
Individual changes in mRNA expression of vimentin in cervical scrapings from 11 CC patients 24 h after radiation exposure at a TD of 10 Gy. The dotted line indicates the mean value of the changes.

Similar data on postradiation increase in the expression of vimentin at the mRNA level were obtained in the group of 11 patients (Table 2). Thus, the relative expression level of this gene increased by an average of 1.9 times after irradiation at a TD of 10 Gy compared with that before the treatment (Figure 9). As with the proportion of vimentin-positive cancer cells, the change in mRNA expression was inversely proportional to the pretreatment level of *VIM* expression (R = −0.56, *p* = 0.07).

### 2.3. Correlation of Radiation Response of Cervical CSCs with Postradiation Increase in Expression of Vimentin under Experimental and Clinical Conditions

**Figure 10:**
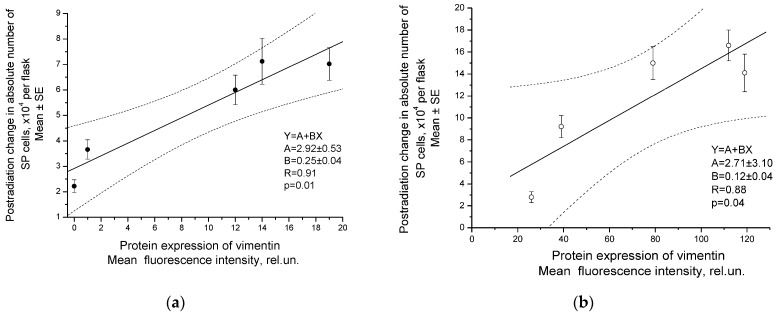
Correlation of postradiation changes in the absolute number of SiHa (**a**) and HeLa (**b**) SP cells with protein expression of vimentin determined by flow cytometry 24 h after fractionated radiation exposure at TDs of 2–10 Gy. Vimentin expression was assessed by the fluorescence intensity of cells stained with antibodies to vimentin after subtracting the mean intensity of nonspecific binding in the control samples. The parameters of the linear regressions Y = A + BX are indicated, where X is vimentin expression and Y is changes in the absolute number of SP cells after irradiation. The dotted lines indicate 95% confidence limits for linear regression (solid line).

Postradiation changes in the absolute number of CSCs (SP cells) correlated with protein expression of vimentin in both cell cultures studied after fractionated radiation exposure at TDs of 2–10 Gy (Figure 10). Statistically significant correlations were found, although no increase in vimentin protein expression was observed after the first dose fractions. The increase in the number of CSCs per unit of vimentin expression (coefficient B of linear regression) was higher in SiHa cells that did not express this protein before irradiation. 

**Figure 11:**
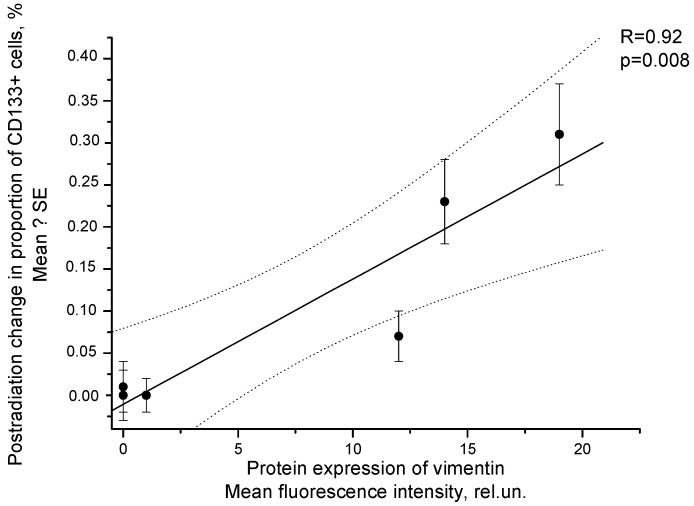
Correlation of postradiation changes in the proportion of CD133^+^ SiHa cells with protein expression of vimentin determined by flow cytometry 24 h after fractionated radiation exposure at TDs of 2–10 Gy. Vimentin expression was assessed by the fluorescence intensity of cells stained with PE-labeled antibodies to vimentin after subtracting the mean intensity of nonspecific binding in the control samples. The dotted lines indicate 95% confidence limits for linear regression (solid line).

A similar correlation was observed between postradiation changes in the number of CD133+ CSCs and the protein expression of vimentin in SiHa cells (Figure 11). In addition, a high correlation was found between the radiation changes in the CSC pool and the mRNA expression of vimentin after irradiation at TDs of 2–10 Gy (R = 0.94, *p* = 0.02).

**Figure 12:**
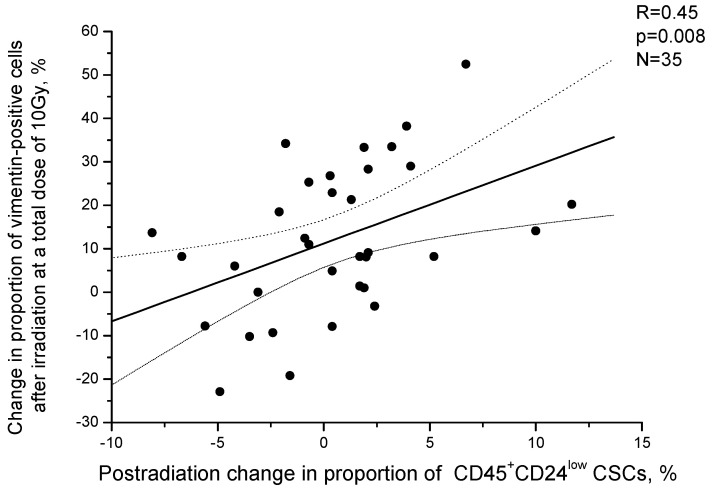
Correlation of changes in the proportion of vimentin-positive cancer cells in cervical scrapings from CC patients with changes in the proportion of CSCs after irradiation at a TD of 10 Gy. Positive values on the X and Y axes indicate an increase in the corresponding indicators after irradiation, whereas negative values indicate a decrease in these indicators compared to those before treatment. The dotted lines indicate 95% confidence limits for linear regression (solid line).

Comparison of vimentin expression with the size of the CSC pool in the cervical scrapings of CC patients showed that the proportion of CSCs before treatment did not depend on the proportion of vimentin-positive cells (R = 0.14, *p* = 0.42). It is important to note that an increase in vimentin expression after irradiation at a TD of 10 Gy correlated with the postradiation increase in the proportion of CSCs observed in some patients (R = 0.45, *p* = 0.008) (Figure 12). A postradiation increase in the CSC proportion was found in 70.4% of patients (19/27) with an increase in vimentin expression after irradiation and only in 25.0% of patients (2/8) with a decrease in the expression of vimentin, i.e., 2.8 times more often (*p* = 0.039 for two-tailed Fisher’s test).

Molecular data on mRNA expression in cervical scrapings of CC patients are in good agreement with this conclusion (Table 2). Thus, the postradiation increase in the relative expression level of *VIM* correlated with the increase in expression of the stemness-related genes studied in our work after irradiation at a TD of 10 Gy, as follows: *SNAIL* (R = 0.87, *p* = 0.0006) and *OCT4* (R = 0.73, *p* = 0.011). In addition, significant associations between the expression of vimentin and the following genes were found in pretreatment samples: *SNAIL* (R = 0.94, *p* = 0.00002), *OCT4* (R = 0.89, *p* = 0.0002), *NANOG* (R = 0.91, *p* = 0.00008).

### 2.4. Prognostic Significance Estimation of Vimentin Expression in Cervical Scraping of CC Patients

The proportion of vimentin-positive cells was compared with the short-term outcome of treatment (degree of tumor regression 3–6 months after radio- or chemoradiotherapy). No differences were found in this indicator either before treatment or after irradiation at a TD of 10 Gy between groups of patients with complete and partial tumor regression. Thus, the proportion of vimentin-positive cells before treatment was, on average, 15.8 ± 2.5% and 12.9 ± 3.7% in the compared groups, respectively; after irradiation—26.1 ± 3.6% and 29.5 ± 4.4%, respectively. There was a tendency to a greater increase in the proportion of vimentin-positive cells after irradiation at a TD of 10 Gy in patients with partial tumor regression than in patients with complete regression. Thus, the average proportion of vimentin-positive cells increased by 16.6 ± 5.4% in 13 patients with partial tumor regression and by only 10.3 ± 3.0% in 22 patients with complete regression (*p* = 0.16). 

A multiple regression analysis was performed to determine the dependence of the tumor regression degree on a number of possible predictors, including the proportion of vimentin-positive cells before and after irradiation at TD of 10 Gy, the change in this indicator after irradiation, disease stage (FIGO), involvement of regional lymph nodes (N+/N0), and histological type (keratinizing/non-keratinizing squamous cell CC). Table 3 shows the parameters of the statistically significant model built using Statistica 6.0 software. The equation included two variables: the stage of the disease (which is quite expected) and the change in the expression of vimentin (with a statistically insignificant correlation coefficient). Thus, by uni- and multivariate analyses, associations at the level of tendency were found between postradiation increase in vimentin expression and unfavorable clinical outcome 3–6 months after treatment. 

It is proposed to find out the significance of vimentin expression in the prognosis of long-term clinical outcomes in the future (overall and disease-free survival of patients).

## 3. Discussion

The resistance of CSCs to a single dose of low-LET ionizing radiation is a well-known phenomenon demonstrated for tumors of experimental animals, human tumor xenografts in immunodeficient mice, and tumor cells of various origins in vitro, including CC cell lines HeLa and SiHa [11,18,19]. This phenomenon can be detected by an increase in the relative or absolute number of CSCs, upregulation of the genes involved in the formation and maintenance of stem-like properties, spheroid growth after irradiation, and more efficient clonogenic survival of irradiated CSCs compared to other cells. Considerably fewer data have been presented on changes in the CSC pool after fractionated radiation exposure in vitro [20,21,22,23,24], and very little data have been obtained regarding the radiation response of the CSCs during radiation therapy of patients [6].

A multiple increase in the CSC absolute number of SiHa and HeLa lines was found in our study after fractionated irradiation at TDs of 2–10 Gy, simulating the first sessions of conditional radiation therapy. As is known, long-term fractionated irradiation at TDs corresponding to the clinical range (50–70 Gy) and above it (90 Gy) can be successfully used to obtain radioresistant cell lines enriched with CSCs [25,26]. A more than 20-fold increase in the proportion of SiHa CSCs was shown after fractionated irradiation at a TD of 30 Gy [27]. A comparison of the effects of single and fractionated irradiation at equal doses on the size of the CSC pool has been performed in a number of studies using cell lines established from glioma and breast cancer [20,22,23]. Fractionated irradiation resulted in a higher enrichment with CSCs, expression of stem-like cell markers, self-renewal related proteins, and activation of Notch-1 signaling pathway as compared to single radiation exposure.

However, in the group of CC patients, we found no significant increase in the average CSC proportion in cervical scrapings after irradiation at a TD of 10 Gy. An increase in the proportion of CSCs was observed in only 57.1% of patients. The reasons for the inconsistency of the data on radiation response of CSCs in established cell lines in vitro and in patients during radiation therapy may be related, first, to much more complicated regulation of pool and properties of CSCs in vivo than those in vitro. As a result of the influence of numerous microenvironmental factors, radiation-induced enrichment with CSCs can decrease in some cases compared with that in vitro, as shown earlier for the yield of CSCs (SP) of murine melanoma line B16 per unit dose [28]. Although the dependencies of proportion of B16 SP cells on dose of γ-irradiation were established both in vitro and in vivo, the increase in the proportion of these cells per dose unit was about 10 times greater in the first case than in the second. Secondly, CSCs represent a heterogeneous population of tumor cells, and various methods identify different CSC subpopulations’ response to radiation, which can vary significantly, as recently shown for breast cancer [29]. Interestingly, the individual radiation response of CD44^+^CD24^low^ CSCs was important for the prognosis and treatment effectiveness of CC patients [6]. Therefore, elucidation of mechanisms for response of this cell population to fractionated irradiation is of interest for further improvement of treatment.

Recently, a profound enrichment with CSCs of several cancer types following exposure to low-LET ionizing radiation has been attributed to not only a higher inherent radioresistance of CSCs and switch from an asymmetric to symmetric type of division during its repopulation, but also radiation-induced reprogramming of non-stem cancer cells into CSC phenotype via EMT. Our results on radiation-induced expression of vimentin in CC cells support the literature data on an increase in the expression level of various EMT markers in cells of cervical and other cancers after fractionated irradiation under experimental conditions [30,31,32,33,34]. A number of authors point to the important role of EMT in the formation of CSC pool [7,8,35,36], and also note the relationship of radiation-induced EMT with an increase in the CSC number and acquisition of stem-like properties [9,11,37,38]. The overwhelming majority of data refer to single irradiation of cell cultures or tumors of experimental animals. The relationship between CSC pool and the EMT process has been much less studied after fractionated radiation exposure simulating regimens of radiation therapy [33,39], and, as far as we know, has not been studied previously in a clinical setting during radiotherapy of cancer patients. Our data on the level of vimentin expression show the relationship between EMT and size of CSC pool (or expression of stemness-related genes) not only under conditions simulating radiation therapy but also during radiation/chemoradiation therapy of CC patients. It is interesting to note that an increase in the absolute number of CSCs occurred after the first session of irradiation of cell cultures at a dose of 2 Gy, while an increase in the level of protein expression of vimentin was found only after irradiation with 4–6 Gy. Therefore, it can be assumed that the contribution of EMT to the radiation increase in the CSC pool becomes noticeable after several dose fractions.

The prognostic value of vimentin expression determined in tumor cells by immunohistochemistry before treatment was previously shown using univariate and multivariate analyses of overall and disease-free survival of patients with squamous cell CC and adenocarcinoma of the uterine cervix [40,41,42,43]. In addition, there have been growing data on the association of other EMT markers, including the transcription factors Snail, Slug, Twist, ZEB1, etc., and EMT-related molecules with poor prognosis of long-term clinical outcome [43,44,45,46]; at the same time, inhibitors or negative modulators of EMT were found to be factors of favorable prognosis of CC patients [47,48]. Taking into account these data, it made sense to analyze the informativeness of the vimentin expression assessment in non-invasive material of scrapings from the cervix before treatment to predict the clinical outcome. However, in contrast to the results of immunohistochemical studies of cancer tissue samples, we did not find a prognostic value of vimentin expression in tumor cells from the pretreatment scrapings, which represent only superficial areas of tumor focus. This negative result is of interest for the choice of material when planning further studies in order to develop predictive biomarkers. It should be noted that the pretreatment proportion of CSCs in cervical scrapings also had no prognostic value for short-term results of radio- and chemoradiotherapy, although the change in this indicator after irradiation at a TD of 10 Gy was an independent predictor of tumor regression degree 3–6 months after the treatment, as we have shown earlier in a group of 38 patients [6]. It could be assumed that the change in the expression of vimentin in the same material would also have predictive value. However, despite the significant correlation of changes in the vimentin expression with the changes in the CSC proportion after irradiation at a TD of 10 Gy (*p* = 0.008), only a tendency to a greater increase in the expression of vimentin was found in patients with partial tumor regression compared to that with complete regression (*p* = 0.16 and *p* = 0.17 in univariate and multivariate analyses, respectively). These results are broadly consistent with the literature data, which indicate an unfavorable value of vimentin expression for prognosis of CC treatment effectiveness. Overall, our results clarify the possibilities and limitations of using a non-invasive method, which is scraping to obtain tumor cells from CC patients for prediction of clinical outcome.

## 4. Materials and Methods

### 4.1. Cell Cultures and Irradiation

HeLa and SiHa cell lines of CC were obtained from the Russian Cell Cultures Collection of the RAS Institute of Cytology (Saint Petersburg, Russia) and the American Type Culture Collection (Rockville, MD, USA). The cells were cultured in Dulbecco’s modified Eagle medium (DMEM) (PanEco, Moscow, Russia), supplemented with 10% fetal bovine serum (FBS) (Biosera, France), penicillin (50,000 U/L, PanEco, Moscow, Russia), streptomycin (50 mg/L, PanEco, Moscow, Russia), and glutamine (292 mg/L, PanEco, Moscow, Russia) in a CO_2_ incubator (Shellab, Cornelius, NC, USA) at +37 °C in a humidified environment containing 5% CO_2_. The cells were passaged twice a week and removed from the substrate with versene and trypsin solutions (PanEco, Moscow, Russia).

After they reached 30–40% confluency, the cells were exposed to fractionated γ-radiation using ^60^Co-based therapeutic facility ‘Rokus-AM’ (Russia) at a single dose of 2 Gy daily until a TD of 10 Gy was reached (the dose rate was 0.8 Gy/min). Three to five independent experiments were performed with each of the cell lines. Absorbed dose determination was carried out in accordance with the International Atomic Energy Agency recommendations set out in TRS-398. A PTW UNIDOS webline with a PTW Farmer^®^ 30010 ionization chamber (PTW, Freiburg, Germany) was used as a dosimeter.

### 4.2. Patients and Treatment

The study group consisted of 46 patients with a histologically confirmed diagnosis of squamous cell CC IB-IIIB stages according to the classification developed by the International Federation of Gynecology and Obstetrics (FIGO). The cervical scrapings from 35 patients were studied at the cellular level to determine the proportions of CSCs and vimentin-positive cells. The samples from 11 other patients were studied at the molecular level to determine mRNA expression of vimentin and stemness-related genes (*NANOG*, *OCT4*, *SOX2*, *SNAIL*, *TWIST*, *CHOP*).

This study was approved by the Ethical Committee of A. Tsyb Medical Radiological Research Center (protocol number 299/2018 from 1 August 2018); all patients signed informed consent for participation in the study.

The mean age of the women was 45.5 ± 1.7 years (from 23 to 76 years). All patients underwent radical courses of specialized treatment at the Department of Radiation and Combined Treatment of Gynecological Diseases (A. Tsyb Medical Radiological Research Center, Obninsk, Russia). Radiation therapy was performed in three patients, and chemoradiotherapy in 43 patients. The treatment in both groups of patients included external irradiation of the primary tumor focus and regional metastasis zones on linear electron accelerator SL-75-5 (Philips, UK) in mode of photon radiation (6 MeV) fractionation at a single focal dose of 2 Gy daily on working days up to a TD of 30 Gy. Then, intracavitary radiation therapy with sources of high ^60^Co activity at a single dose of 5.0 Gy 2 times per week was performed until TD reached 35–40 Gy. The dose rate was 3.5–5.0 Gy/min for external irradiation, and 0.3–0.4 Gy/min for intracavitary irradiation. The patients undergoing chemoradiotherapy were administered concurrently intravenous infusions of cisplatin, 40 mg/m^2^, every week during a period of external beam irradiation. The first infusion of cisplatin was performed 3–4 h before the first radiotherapy session. Subsequent infusions of cisplatin were also carried out 3–4 h before irradiation.

The degree of tumor regression was determined 3–6 months after completion of the treatment according to the results of clinical and radiological examination (rectovaginal examination, ultrasound, magnetic resonance imaging, etc.) in accordance with RECIST recommendations (version 1.1) [49].

### 4.3. CSC Identification in Cell Cultures and Cervical Scrapings from CC Patients

**Figure 13:**
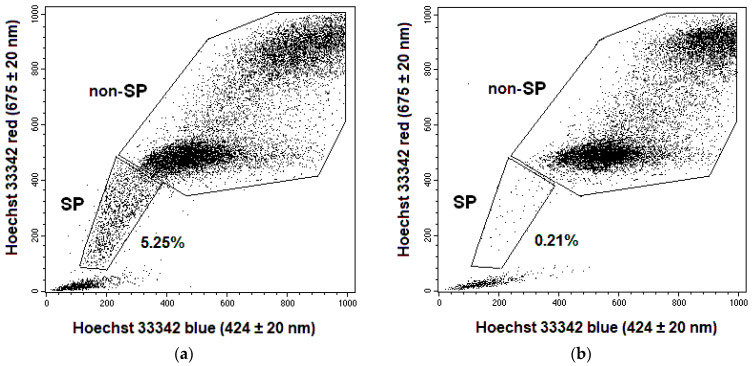

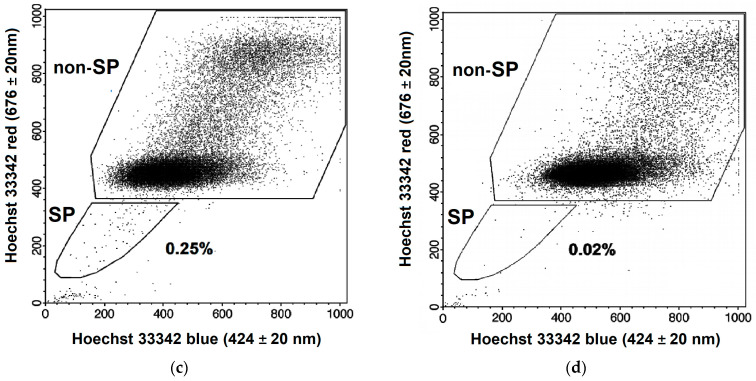
Representative dot plots for Hoechst blue vs. Hoechst red simultaneous emission of HeLa (**a**,**b**) and SiHa (**c**,**d**) cells in unexposed samples without (**a**,**c**) or with the addition of verapamil (**b**,**d**). The regions of SP and non-SP cells were selected for sorting, and the proportion of SP cells was determined.

For SP assay, HeLa and SiHa cells were removed from flasks using the versene/trypsin solution (1:1) into serum-free DMEM at a dilution of 1 million cells/mL 24 h after irradiation at TDs of 2, 4, 6, 8, and 10 Gy. Some samples were incubated for 15 min at +37 °C with verapamil (12.5 mg/mL) (EBEWE Pharma, Unterach, Austria), which is an ATP-binding transporter blocker preventing reverse transport from cells of a number of substances, including Ho342. Then, 10^6^ cells with and without verapamil were stained with Ho342 dye (Merck, Darmstadt, Germany) at a concentration of 5 µg/mL during 90 min at +37 °C. After incubation, the cells were centrifuged at 250× *g*, the sediment was resuspended in cold Hanks’ solution (PanEco, Moscow, Russia), containing 2% FBS, 10 mM Hepes (PanEco, Moscow, Russia), and 2 µg/mL propidium iodide (PI) (Sigma-Aldrich, Burlington, MA, USA). The samples were analyzed and sorted using FACS Vantage (Becton Dickinson, San Diego, CA, USA). Ho342 fluorescence was measured in red (675 ± 20 nm) and blue (424 ± 20 nm) spectral intervals at λ_excitation_ of 364 nm. PI fluorescence was measured in a range of 585 ± 20 nm at λ_excitation_ of 488 nm. SP was identified among PI^−^ cells which were selected after the routine scatter gate procedure (Figure 13). The samples with verapamil served as a negative control, in which the proportion of SP cells was significantly reduced. SP and non-SP cells were sorted on glass slides from unexposed samples without verapamil for further analysis of vimentin expression. The absolute number of SP cells was established by multiplying the proportion of these cells by the total quantity of cells in a flask.

The SiHa cells were removed from the flasks into cold Hanks’ solution and incubated with monoclonal antibodies to CD133 labeled with phycoerythrin (PE) (BD, CA, USA) at a concentration of 20 µL per 1 million cells. Isotype control antibodies to snail hemocyanin conjugated with the same fluorochrome (BD, CA, USA) were used to evaluate nonspecific binding. The specimens were incubated with antibodies for 30 min on ice in darkness, then washed in PBS (pH 7.2), and analyzed using FACS Vantage. The proportion (%) of CD133^+^ cells was determined among undamaged cells gated by forward and side light scattering.

**Figure 14:**
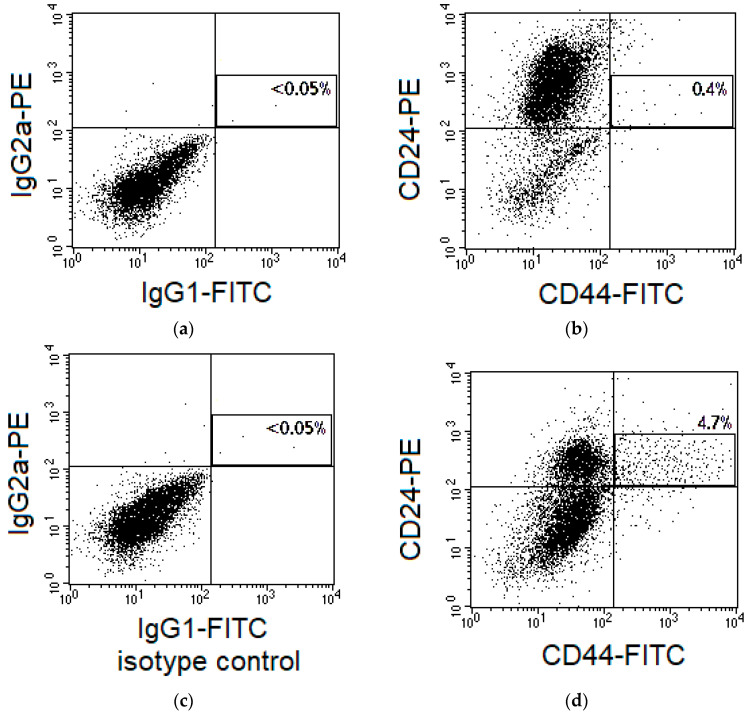
Representative dot plots for fluorescence of cervical cells from two patients with CC in the control of nonspecific binding (**a**,**c**) and after staining with antibodies to CD44 and CD24 (**b**,**d**) labeled with fluorescein isothiocyanate (FITC) and PE. Cases with different expressions of CD24 in the main part of cells and different proportion of CD44^+^CD24^low^ cells are presented after gating for CD45^−^ Hoechst33342 (Ho342)^+^ events. The region of CD44^+^CD24^low^ cells is highlighted, and their percentage among CD45^-^Ho342^+^ events is indicated.

Cervical scrapings were collected before treatment and 24 h after irradiation at a TD of 10 Gy. In the case of chemoradiotherapy, cisplatin was injected before the first radiation session, as described above. The material was placed in tubes containing a complete DMEM (Paneco, Moscow, Russia) and transported to the laboratory within one hour at room temperature. Next, the cell suspension was obtained, nucleated cells were counted using a Goryaev’s chamber, and 200,000–300,000 cells were aliquoted. CSCs were identified by immunophenotyping using a four-color flow cytometric analysis with FACS Vantage (BD, CA, USA). The cell suspensions were stained using monoclonal antibodies labeled with different fluorochromes to the following surface markers: CD44 (clone L178 binding various CD44 isoforms), CD24, and CD45 according to the standard instructions by the manufacturer (BD, CA, USA). Ho342 was added to the stained samples at a concentration of 6 µg/mL 10 min before flow cytometric analysis. The markers for the identification of cervical CSCs were selected on the basis of the published data on high CD44 expression and low CD24 expression on the surface of these cells [50,51]. Staining with antibodies to CD45 was performed for negative selection of lymphoid cells. Binding with Ho342 was used for positive selection of nucleated cells and negative selection of debris and erythrocytes. The nonspecific background signal was differentiated from the specific antibody signal by an isotype control using monoclonal antibodies of the corresponding isotype to limpet hemocyanin conjugated with the same fluorochromes as antibodies to the specific surface markers (BD, CA, USA). The proportion (%) of CD44^+^CD24^low^ CSCs was estimated among CD45^−^Ho342^+^ nucleated cells. The region of CD44^+^CD24^low^ cells was selected as shown in Figure 14, taking into account the control of nonspecific binding and variability of CD24 expression in the samples from various patients.

### 4.4. Determination of Vimentin Expression at Protein Level in Cell Cultures and Cervical Scrapings from CC Patients by Flow Cytometry, Fluorescent Microscopy, and Laser Scanning Confocal Microscopy 

The influence of low-LET radiation exposure in the conventional fractionation regime on protein expression of vimentin in the total cell population was studied using flow cytometry (in HeLa and SiHa cell cultures) and fluorescent microscopy (in cervical scrapings from 35 CC patients).

The HeLa and SiHa cells were fixed in cold acetone 24 h after irradiation at TDs of 2, 4, 6, 8, and 10 Gy and stained with monoclonal antibodies to vimentin labeled with PE (BD Biosciences, CA, USA) at a ratio of 20 µL antibodies/1 million cells for 1 h, according to the manufacturer’s instructions. Isotype control monoclonal antibodies to snail hemocyanin, conjugated with PE (BD Biosciences, USA), were used to check nonspecific binding. The cells were washed from unbound antibodies in PBS (pH 7.2) and immediately analyzed using FACS Calibur (BD, CA, USA) for intensity of forward and side light scattering and PE fluorescence. CellQuestPro software was used to establish the mean PE fluorescence intensity of cells after the routine scatter gate procedure.

Cervical scrapings were used to prepare smears on the glass slides. Vimentin expression was determined in cancer cells 24 h after irradiation at a TD of 10 Gy using fluorescent microscopy followed by acetone fixation at −20 °C for 20 min and intracellular staining with monoclonal antibodies to vimentin labeled with PE (BD Biosciences, CA, USA) at a 1:50 dilution at room temperature for 1 h. The cells were washed twice from unbound antibodies in PBS and placed into MOUNT-QUICK medium (Daido Sangyo Co., Ltd., Tokyo, Japan). Isotype control monoclonal antibodies to snail hemocyanin, conjugated with PE (BD Biosciences, CA, USA), were used to check nonspecific binding. Analysis of the stained samples was carried out on an Optiphot-2 fluorescence microscope (Nicon, Tokyo, Japan) with 40 × objective using a G-2 A filter combination (λ_excitation_ = 510–560 nm, λ_emission_ > 590 nm). The lymphocytes in cervical scrapings were an internal positive control of immunocytochemical reactions with antibodies to vimentin. The proportion of vimentin-positive cancer cells was determined among all cancer cells. 

Vimentin expression was evaluated in sorted SP and non-SP cells of the HeLa line using confocal laser scanning microscopy. The cells were fixed on glass slides by acetone at −20 °C for 20 min, and then incubated with vimentin polyclonal goat antibodies labeled with PE (Santa Cruz Biotechnology, Dallas, TX, USA) for 40 min, according to the manufacturer’s instructions. Thereafter, the cells were washed twice from unbound antibodies in PBS and placed into MOUNT-QUICK medium (Daido Sangyo Co., Ltd., Japan). Goat immunoglobulins labeled with PE (R&G Systems, North Port, FL, USA) were used as control of nonspecific binding. The samples were analyzed using the confocal laser scanning microscope TCS SPE 4000 (Leica Microsystems, Wetzlar, Germany) with spectral detection by a multi-channel analyzer. A solid laser with a wavelength of 488 nm was used for fluorescence excitation; fluorescence intensity was assessed within a range of 550 to 610 nm. Subsequent processing of the obtained images was carried out using Imaris 7.2.3 software (Bitplane AG, Schlieren, Switzerland). The mean intensity of cell fluorescence per pixel was determined and used to evaluate the level of vimentin expression. At least 200 SP and 200 non-SP cells were analyzed in each sample.

### 4.5. Polymerase Chain Reaction for Detection of mRNA Expression of Vimentin and Stemness-Related Genes

Total RNA was extracted from SiHa cells 24 h and 8 days after irradiation at TDs of 2, 4, 6, 8, and 10 Gy and from the cervical scrapings of 11 CC patients before treatment and 24 h after irradiation at a TD of 10 Gy. RNAzol^®^ RT RNA Isolation Reagent (Sigma Aldrich, Burlington, MA, USA) was used according to the manufacturer’s protocol. The quality and quantity of RNA were evaluated using the NanoDrop ND-1000 Spectrophotometer (NanoDrop Technologies Inc., Wilmington, DE, USA) and using electrophoresis by 28S/18S rRNA ratio. Reverse transcription was performed using the High-Capacity cDNA Reverse Transcription Kit (Applied Biosystems, Bedford, MA, USA) with 500 ng of total RNA per reaction immediately after RNA isolation. All the cDNA samples were stored at −20 °C until qPCR analyses. The primer sequences used for PCR (Table 4) were designed using the Primer-BLAST NCBI Online Tool [52]. PCR amplification and fluorescence detection were performed using qPCRmix-HS SYBR (Eurogene, Russia) on the Rotor Gene Real-Time PCR detection system (Corbett Research, Mortlake, Australia). A melting curve was used to monitor the specificity of the primer. The threshold cycles were determined using Rotor Gene software. Relative levels of mRNA expression were determined using the ΔCt method against reference. Fold changes in expression levels were determined in irradiated SiHa cells and cervical samples using the ΔΔCt method. In this case, the postradiation expression level of each gene was normalized against reference in the same sample and then against the expression level (normalized to reference) of this gene in the pretreatment sample of the same patient or control samples of unirradiated SiHa cells. The references were selected using the RefFinder service [53] among five candidate genes: *ALAS1*, *GAPDH*, *GUSB*, *IPO8*, and *YWHAZ*. Based on expression stability, the *ALAS1* housekeeping gene was chosen as a reference gene for experiments in vitro; *GAPDH* and *IPO8* were chosen as reference genes for cervical scrapings. All experiments were conducted in triplicate.

### 4.6. Statistical Analysis

Statistical processing of the results was performed using the programs Origin 6.0 (Microcal Software, Inc., Northampton, MA, USA) and Statistica 6.0 (StatSoft, Inc., Tulsa, OK, USA). For descriptive statistics of quantitative characteristics, average values and standard errors (SE) were used. Correlation analysis was performed to assess the relationship between the two quantitative parameters. The groups were compared using the Mann-Whitney and Fisher criteria. Differences between the groups were considered statistically significant at *p* < 0.05. 

## 5. Conclusions

There is great interest in the elucidation of the patterns and mechanisms of the cervi-cal CSC response to fractionated radiation exposure under experimental conditions in vitro simulating regimens of conventional radiotherapy and, especially, under clinical conditions during the treatment of patients. Collectively, our findings have shown that fractionated low-LET radiation at TDs up to 10 Gy causes an increase in the absolute number of cervical CSCs in well-known established cell lines and an increase in the relative number (proportion) of these cells in the cervical scrapings from a part of CC patients. Postradiation changes in the CSC pool significantly correlated with changes in the expression of vimentin in cell cultures in vitro and clinical samples, despite different methods for detecting CSCs (SP, immunophenotyping by CD133, CD44, CD24) and assessing vimentin expression (flow cytometry, PCR, immunocytochemistry). These findings elucidate some of the relationships between EMT, CSCs, and therapeutic resistance that are needed to develop new strategies for cancer treatment in the future.

## Figures and Tables

**Table 1:** Comparison of the proportion of vimentin-positive cancer cells with different clinical and morphological parameters of disease and treatment.

Clinical, Morphological Parameters and Treatment		Number of Patients	Proportion of Vimentin-Positive Cells, % Average Value ± SE		
			Before Treatment	After Radiation Exposure at a TD of 10 Gy	Postradiation Changes
FIGO stage	I	6	17.4 ± 6.4	38.6 ± 9.4	20.2 ± 6.0
	II	18	14.6 ± 3.0	24.2 ± 3.3	9.6 ± 3.1
	III	11	13.5 ± 3.3	27.4 ± 4.9	13.9 ± 6.6
Status of lymph node involvement	N0	23	14.8 ± 2.4	29.0 ± 3.7	14.2 ± 3.8
	N+	12	14.5 ± 4.2	24.4 ± 4.0	9.9 ± 3.9
Histological type of squamous cell CC	Keratinizing	7	12.2 ± 3.7	19.5 ± 5.1	7.3 ± 5.8
	Nonkeratinizing	28	15.5 ± 2.5	29.8 ± 3.1	14.3 ± 3.2
Treatment	Radiotherapy	3	27.2 ± 9.5	37.6 ± 10.3	10.4 ± 9.3
	Chemoradiotherapy	32	13.6 ± 2.0	25.9 ± 2.7	12.3 ± 3.0

**Table 2:** Relative levels of mRNA expression of vimentin (*VIM*) and a number of stemness-related genes before treatment and after irradiation at a TD of 10 Gy against reference (*GAPDH* and *IPO8*).

Patient Number	Total Dose, Gy	Gene						
		*CHOP*	*NANOG*	*OCT4*	*SNAIL*	*SOX2*	*TWIST*	*VIM*
1	0	0.26	7.74	64.97	1.16	0.03	0.03	110.80
	10	0.04	8.01	72.63	0.18	0.02	0.02	78.93
2	0	0.50	35.86	105.02	6.80	0.03	0.16	316.16
	10	0.56	5.26	83.64	9.82	0.01	0.05	243.21
3	0	0.48	3.85	46.35	2.76	0.01	0.16	88.32
	10	0.54	38.65	70.63	7.37	0.27	0.72	170.34
4	0	0.02	9.18	2.79	0.14	0.24	0.50	66.16
	10	0.03	8.31	10.23	0.83	0.30	0.89	150.55
5	0	0.03	7.68	4.93	0.51	0.64	0.13	9.85
	10	0.42	10.48	62.68	1.66	0.08	0.48	22.78
6	0	0.02	8.85	4.81	0.16	0.30	0.24	26.45
	10	0.23	12.29	143.00	3.12	0.02	0.54	120.25
7	0	0.02	9.08	3.17	0.02	0.13	0.25	13.95
	10	0.05	14.76	7.28	0.14	0.12	0.18	48.96
8	0	0.04	12.94	4.12	0.14	0.04	0.72	74.75
	10	0.14	17.11	7.10	0.12	0.19	0.43	59.17
9	0	0.10	5.97	1.49	0.20	1.00	0.03	19.00
	10	0.14	7.12	1.95	0.53	0.56	0.09	45.92
10	0	0.03	3.78	4.56	0.47	0.08	0.26	19.29
	10	0.04	9.28	9.81	0.36	0.05	0.17	27.74
11	0	0.30	4.46	8.50	1.07	0.12	0.47	30.42
	10	0.09	12.65	5.98	1.30	0.06	0.15	9.99

**Table 3:** The results of multiple regression analysis of the dependence of tumor regression degree on disease stage and postradiation change in vimentin expression.

Indicator (Predictor)	Beta ^1^	*p*-Level for Predictor	R	*p*-Level for Model in the Whole
Disease stage (FIGO)	0.38	0.03	0.44	0.049
The change in the proportion of vimentin-positive cells after irradiation at a TD of 10 Gy	0.18	0.17		

^1^ Beta is the standardized angular regression coefficient (in SD units), R is the multiple correlation coefficient.

**Table 4:** Gene-specific primers used for real-time polymerase chain reaction (PCR).

Gene	5′→3′ Sequence (F–Forward, R–Revers)	PCR Product Size, bp
*ALAS1*	F: TGCTGCAAAGATCTGACCCCTC	113
	R: AAACTCATGGGCCACATCACAC	
*GAPDH*	F: CCTCTGACTTCAACAGCGACA	101
	R: GTTGTCATACCAGGAAATGAGCTTG	
*GUSB*	F: GCGTAGGGACAAGAACCACC	120
	R: TCCAAGGATTTGGTGTGAGCG	
*IPO8*	F: GCAGAGTGTCATGCAGCTAAAC	120
	R: GACCCCTCGAGTTAATCTCTCCA	
*YWHAZ*	F: TGGTGATGACAAGAAAGGGATTGT	119
	R: AGTTAAGGGCCAGACCCAGT	
*VIM*	F: GAGATGCTTCAGAGAGAGGAAGCC	112
	R: CTTGCAAAGATTCCACTTTGCGT	
*NANOG*	F: GATGCCTCACACGGAGACTG	108
	R: GCAGAAGTGGGTTGTTTGCC	
*OCT4*	F: AGGAGAAGCTGGAGCAAAACC	81
	R: GGAGCTTGGCAAATTGCTCG	
*SOX2*	F: CATGAAGGAGCACCCGGATT	124
	R: CCCGCTCGCCATGCTATT	
*SNAIL*	F: AGCGAGCTGCAGGACTCTAA	125
	R: CAGATGAGCATTGGCAGCGA	
*TWIST*	F: GGCCGGAGACCTAGATGTCATT	150
	R: CACGCCCTGTTTCTTTGAATTTGG	
*CHOP*	F: ACCTCCTGGAAATGAAGAGGAAGA	111
	R: GAGGTGCTTGTGACCTCTGC	

## Data Availability

The data that support the findings of this study are available from the corresponding author upon reasonable request.
